# *Plasmodium vivax* malaria in Mali: a study from three different regions

**DOI:** 10.1186/1475-2875-11-405

**Published:** 2012-12-05

**Authors:** Maria Bernabeu, Gloria P Gomez-Perez, Sibiri Sissoko, Mohamed B Niambélé, Allassane Ag Haibala, Ariadna Sanz, Mahamadou A Théra, Carmen Fernandez-Becerra, Klénon Traoré, Pedro L Alonso, Quique Bassat, Hernando A del Portillo, Ogobara Doumbo

**Affiliations:** 1 Barcelona Centre for International Health Research (CRESIB, Hospital Clínic-Universitat de Barcelona), Barcelona, 08036, Spain; 2 Malaria Research and Training Centre, Department of Epidemiology of Parasitic Diseases, Faculty of Medicine, Pharmacy and Dentistry, UMI-Mali 3189, University of Sciences, Techniques and Technologies, Bamako, Bamako, B.P. 1805, Mali; 3 Programme National de Lutte Contre le Paludisme, Ministère de la Santé, Bamako, B.P. 232, Mali; 4 Institució Catalana de Recerca i Estudis Avançats (ICREA), Barcelona, 08010, Spain

**Keywords:** Mali, Sub-Saharan Africa, Plasmodium vivax, Vivax malaria, Nested-PCR, DNA sequencing, SSU RNA, Giemsa-smears

## Abstract

**Background:**

*Plasmodium vivax* has traditionally been considered virtually absent from Western and Central Africa, due to the absence of the Duffy blood group in most of the population living in these areas. Recent reports, however, suggest the circulation of *P. vivax* in sub-Saharan Africa.

**Methods:**

Giemsa/Field-stained smears from febrile patients recruited in five different cities (Goundam, Tombouctou, Gao, Bourem and Kidal) pertaining to three regions from Northern Mali were examined. Nested-PCR and DNA sequence analyses of selected samples were performed to fully confirm the presence of *P. vivax* infections.

**Results:**

Results demonstrated the presence of *P. vivax* infections in close to 30% of the cases as detected by Giemsa/Field-stained smears and nested-PCR and DNA-sequence analyses of selected samples unequivocally confirmed the presence of *P. vivax*.

**Conclusions:**

The diagnostics of this human malaria parasite should be taken into account in the context of malaria control and elimination efforts, not only in Mali, but also in sub-Saharan Africa.

## Background

*Plasmodium vivax* is the most widely distributed human malaria parasite and responsible for 100–300 million clinical cases yearly, including severe disease and death. It has been estimated that 2.85 billion people are exposed to some risk of *P. vivax* transmission worldwide and of those circa 0.10 billion (3.5%) are in the African region where transmission is considered stable (≥0.1 case per 1,000 people per annum) [1]. Of note, it is widely accepted that *P. vivax* is largely absent in Central and Western Africa, mostly as a result of the absence of the Duffy blood group, a blood receptor hitherto considered indispensable for the invasiveness of *P. vivax* into red blood cells, as more than 90% of the population in this area are believed to be Duffy negative [2]. In recent years, however, increasing evidence of the circulation of *P. vivax* episodes affecting Duffy negative individuals has appeared, including one from Africa [3-5]. Ever since, other reports have demonstrated the presence of *P. vivax* infections in sub-Saharan Africa [6-9].

Mali is a landlocked nation in West Africa divided into eight regions and almost 15 million people with international borders with seven African countries. Of relevance, some reports have indicated the presence of *P. vivax* infections in this country [10-12]. Here, following a survey in the northern population of Mali, where local transmission of *P. vivax* was also suspected, blood samples were collected from febrile patients and stained. Giemsa/Field-stained blood smears were analysed for specific *P. vivax* diagnosis by microscopy. Moreover, genomic DNA extracted from the same smears used in microscopy was used as templates for nested-PCR with primers for the four major *Plasmodium* species infecting humans. Last, amplified fragments were cloned and sequenced. Results unequivocally confirm and extend previous observations of *P. vivax* in Mali.

## Methods

### Samples collection and microscopy diagnosis

**Figure 1:**
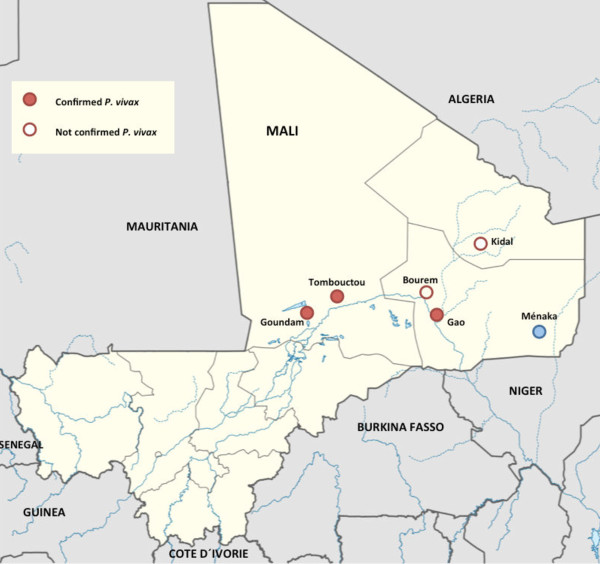
**Vivax malaria in Mali.** Geographical regions where this study was conducted are circled in red. Ménaka, the geographical area where *P. vivax* was also recently detected [10] is circled in blue. Adaptation from the map produced by NordNordWest [CC-BY-SA-3.0-de (http://creativecommons.org/licenses/by-sa/3.0/de/deed.en)], via Wikimedia Commons.

As part of routine National Malaria Control Programme surveillance procedures, Giemsa and/or Field-stained thick and thin blood smears from three different regions of Mali: Tombouctou, Gao and Kidal were collected in five health care facilities within these regions (Figure 1). They were afterwards collected and sent to the Malaria Research and Training Centre in Bámako to assess the quality of microscopy diagnosis and survey the malaria incidence. Samples belonging to 88 febrile patients from these health care facilities were randomly chosen and re-examined in a centralized manner at the Barcelona Centre for International Health Research laboratories (Barcelona, Spain) following the World Health Organization’s operational manual 2011 for universal access to malaria diagnostic testing. The microscopic diagnosis of these specimens performed in Mali was not available to researchers in Barcelona with the exception of one sample. Therefore, blood smears were studied blindly assessing the presence or absence of parasites with morphological features of *P. vivax* mature stages using a Nikon ECLIPSE 50i microscope. Pictures were taken with a Nikon photomicrography camera model DS-5M. Smears were reported as negative after the examination of the specimen in approximately 200 oil immersion fields, and as positive when at least one parasite with highly suggestive features of *P. vivax* mature stages was observed.

### Genomic extraction

Genomic DNA from 25 smears pertaining to different patients was obtained by scrapping off half of the surface of the smear with a scalpel and re-suspending in 100 μl of phosphate buffered saline (PBS). Genomic DNA was extracted using QIAamp DNA mini kit (Qiagen) according to manufacturer’s instructions.

### PCR diagnosis and sequencing

Identification of the four species was performed by nested-PCR [13]. *Plasmodium vivax* positive agarose bands were extracted using QIAEX II Gel Extraction Kit (Qiagen) and cloned using TOPO TA Cloning Kit and pGEM-T Easy Vector Systems. Cloned plasmids were sequenced using commercial primers FM13 and T7. Sequence alignment and phylogenetic trees were performed using Lasergene software (DNASTAR Corp., Madison, WI). *Plasmodium spp* small subunit rRNA (SSU RNA) sequences were obtained from PlasmoDB version 8.2 and GenBank. *Plasmodium vivax*: PVX_096002, PVX_097020, PVX_088869, PVX_079693; *Plasmodium cynomolgi*: FJ619084.1; *Plasmodium falciparum*: JQ627152.1; *Plasmodium knowlesi*: FJ619098.1; *Plasmodium malariae*: GU815531.1; *Plasmodium ovale*: JF894411.1; *Plasmodium yoelii*: AF266261.1. Sequences were analysed by the parsimony method using 100 heuristic random addition replicatives and 100 bootstrap replicates.

## Results

**Figure 2:**
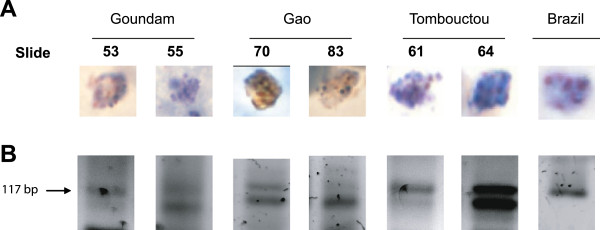
***Plasmodium vivax *infections in Northern Mali. A**. Giemsa-stained images of samples from different regions. **B**. Amplified fragments from a nested-PCR assay using *P. vivax-*specific primers. Molecular weight in base pairs (bp) of predicted amplified size fragments (117 bp) corresponding to *P. vivax* (arrow) is shown to the left.

A total of 88 Giemsa/Field-stained slides were randomly selected from febrile malaria patients as part of routine National Malaria Control Programme surveillance procedures in Mali. Strikingly, out of 88 samples analysed, 25 (28.4%) contained mature stages (readily detected in less than 20 high power fields examined) suggestive of *P. vivax* (Figure 2A, Additional file 1, Table 1). As *P. ovale* and *P. malariae* infections are commonly found in Western and Central Africa, genomic DNA was extracted from the 25 slides for *P. vivax* and nested-PCR analysis was performed using probes for the four *Plasmodium spp* (Figure 2B). As a positive control, gDNA from a *P. vivax* slide from Brazil was also extracted. Fifteen samples pertaining to Goudam, Tombouctou and Gao were PCR positive for *P. vivax*. Among them, eleven contained mixed infections with *P. falciparum*, four were positive for *P. malariae* and none for *P. ovale* (Table 2).

**Table 1:** Total numbers and analyses of samples obtained from five different cities in the North of Mali

**Cities**	**Samples**	**Microsocopy Pv+/examined**	**PCR +**	**Sequencing**
**Bourem**	**20**	**1/20**	**0/1**	**ND**
**Gao**	**46**	**7/12**	**5/7**	**5/15**
**Goundam**	**22**	**3/8**	**3/3**	**3/15**
**Kidal**	**34**	**5/30**	**0/5**	**ND**
**Tombouctou**	**58**	**9/18**	**7/9**	**3/15**
**Total**	**180**	**25/88**	**15/25**	**11/15**

**Table 2:** **Nested-PCR identification of *Plasmodium spp. *in 25 samples from five different cities pertaining to three regions in Northern Mali**

**Cities**	***P. falciparum***	***P. vivax***	**Mixed *P. falciparum *+ *P. vivax***	**Mixed *P. falciparum *+ *P. malariae***	**Mixed *P. falciparum *+ *P. vivax *+ *P. malariae***
**Bourem**	*1/1*	*0/1*	0/1	0/1	0/1
**Goundam**	*0/3*	*0/3*	3/3	0/3	0/3
**Tomboctou**	*0/9*	*4/9*	1/9	1/9	2/9
**Kidal**	*4/5*	*0/5*	0/5	0/5	0/5
**Gao**	*1/7*	*0/7*	5/7	1/7	0/7
**Total**	*6/25*	*4/25*	9/25	2/25	2/25

**Figure 3:**
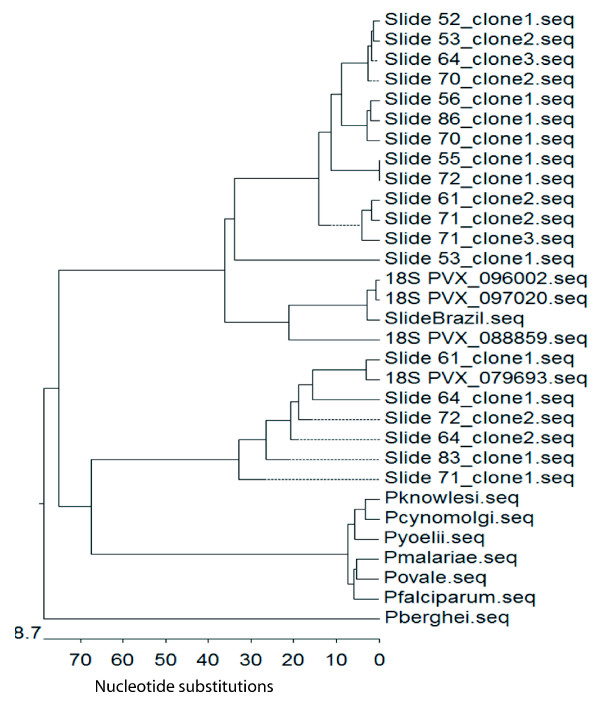
**Bootstrapped phylogenetic rooted tree constructed by the parsimony method for the SSUr RNA sequences of *Plasmodium vivax *from Mali.** Horizontal branch lengths between nodes correspond to the number of shared derived changes. Slide52-72_clone1-3 represent the slide from Mali were we obtained the gDNA and the number of clone that was sequenced. SlideBrazil, positive *P. vivax* control. 18S_PVX samples were obtained from PlasmoDB 8.2. Other *Plasmodium spp.* 18S sequences were obtained from GenBank. Accession Numbers for all of them are listed in Methods.

To fully confirm these results, amplified fragments from eleven samples were cloned and sequenced. Significantly, similarity analysis with available *P. vivax* SSU RNA gene sequences from the SalI strain (PVX_088869, PVX_096002, PVX_097020, PVX_079693) demonstrated that sequences from Mali corresponded to *P. vivax* and that there were several polymorphisms (Additional file 2). Consistent with the existence of different SSU RNA genes in *P. vivax*[14], phylogenetic analysis tested by bootstrap showed that sequences fell into major genetic clusters of SSU RNA *P. vivax* genes branching-out from SSU RNA sequences of other *Plasmodium* species (Figure 3). Together, these results unequivocally confirmed the presence of *P. vivax* infections in Mali.

## Discussion

The presence of *P. vivax* malaria in sub-Saharan Africa has been largely neglected since it was demonstrated that lack of expression of the Duffy blood-group correlated with the absence of *P. vivax* infections [2]. Moreover, it has been suggested that *P. vivax* is of African origin thus driving fixation of the Duffy negative phenotype [15]. Recent evidence, however, has called upon to revise this suggestion as it strongly indicates that *P. vivax* may instead be of Asian origin [16,17]. Its increasingly recognized presence in Africa may, therefore, be related to its newly acknowledged capacity of exploiting new alternative invasion pathways among populations in this continent. Regardless of the geographical origin of *P. vivax* and the pathway for entrance into reticulocytes, it is clear that the presence of *P. vivax* in Africa is probably underestimated.

A few reports, of variable quality, have described infections by *P. vivax* in Mali [10-12]. An early indication of its presence came from the description of Russian doctors in the region of Gao in the 1950s (OD, unpublished). Four decades later, a case of *P. vivax* infection in an eight year-old girl was reported in Kidal during a survey in 1988 in Trans-Sahara [12]. The microscopy diagnosis of this patient was confirmed in two European reference laboratories at Marseille and London. More recently, Koita *et al.* performed two cross-sectional studies in the North-Eastern region of Mali, reporting over 10% prevalence rates of *P. vivax* infections as detected by Giemsa-stained smear [10]. However, and noteworthy, with the exception of one single sample, exclusion of the potentially confounding and morphologically similar *P. ovale* and *P. malariae* species were not systematically performed using molecular techniques.

Microscopy evaluation of samples examined here similarly suggested the presence of parasite blood stages resembling *P. vivax*. However, and as an important limitation of only using such diagnostic techniques, it seems difficult to exclude that these stages may not correspond to *P. ovale* species. *P. ovale* has been frequently reported in this particular region of Africa and even though distinct ameboid trophozoites and number of nuclei per single schizont can be used as criteria for species-specific diagnostics, this remains very challenging in most thick-blood smears. Thus, nested-PCR and DNA sequence analyses were performed to unambiguously demonstrate *P. vivax* infections in Mali. Amplified fragments of sizes corresponding to the SSU RNA gene of *P. vivax* were observed in close to 28% of infections. Moreover, sequence similarity and phylogenetic analyses unequivocally confirmed that these fragments corresponded to *P. vivax* sequences.

These findings are of important public health relevance. In recent years, the scientific attention of the malaria community has shifted from a focus on malaria control to specific efforts aiming at global malaria eradication [18]. However, it is important to consider that efforts needed to control *P. vivax* will surely exceed those necessary to control *P. falciparum* as transmission of this species is possible even before the appearance of clinical symptoms, and can also occur irrespective of adequate asexual parasite clearance as a result of hypnozoite-derived relapses. Indeed, the adequate treatment of *P. vivax* infections includes the addition of a full 14-day long course of primaquine (PQ), the only to date registered drug that can achieve the radical cure of the hepatic hypnozoites, responsible for unpredictable relapses and subsequent morbidity, and a drug that has potent gametocytocidal activity. Without the radical cure, hepatic hypnozoites will maintain those infected individuals as infectious, and thus prone to maintain transmission and develop new disease episodes. PQ is unfortunately an unsafe drug, and can lead to severe haemolysis when administered blindly to glucose-6-phosphate dehydrogenase (G6PD) deficient patients, a genetic deficiency particularly frequent in sub-Saharan Africa, limiting therefore its widespread use unless guaranteeing prior screening of this deficiency. As no rapid G6PD deficiency diagnostic tests exist to date, dismissing its risks prior to PQ administration appears unfeasible, and adequate treatment of *P. vivax* episodes unguaranteed, adding further complexity to the control programmes in place in many African settings. In Mali, the spread of *P. vivax* in the northern part of the country will complicate the possibility of malaria elimination. The current malaria control programme has no reference to vivax malaria. The findings reported here will push further to a revision of the policy document. New tools will be needed (the use of mass drug administration of primaquine for example) and access to the target population is a major public health issue. All the laboratory technicians in this endemic region of *P. vivax*, should be retrained for microscopic diagnostic.

An important limitation of this work is the inability to link such confirmed *P. vivax* infections with the Duffy and G6PD phenotypes of the individuals affected. Indeed, the presence of Duffy positive ethnic groups in central and West Africa as in the case of the Moors in Mauritania [8] or other Ethnic groups that may be present in Mali could entirely account for *P. vivax* transmission in the area, and would not necessarily imply that this species has found alternative mechanisms to infect Duffy-negative individuals. Prospective studies further investigating the Duffy and G6PD status and its relation to species-specific malaria risk are, therefore, urgently needed.

In Mali, *P. vivax* infections can no longer be considered rare or anecdotal, and their diagnosis and adequate management need to be included as part of routine surveillance activities. This should be coupled with an adequate knowledge of the Duffy and G6PD status in the population, so as to assess the risks that the introduction of PQ treatment could entail to the population. Malaria control efforts in the area will surely fail unless species-specific measures are put in place.

## Abbreviations

G6PD: Glucose-6-phosphate dehydrogenase; PQ: Primaquine; SSU RNA: Small subunit rRNA.

## Competing interests

The authors declare that they have no competing interests.

## Authors’ contributions

MB and GGP performed the experiments. MB, GGP, CFB, HAP analysed data. AS, MTHA managed the project. SS, AH, MBN, KT, MTA did the samples collection. QB, PLA, HAP and OD conceived and designed the study. HAP, QB, MB, GGP and OD wrote the paper. All authors read and approved the final manuscript.

## Supplementary Material

Additional file 1 **Vivax malaria in Mali. Additional illustrative images of Giemsa-stained smear *P. vivax *positive samples from different regions.** Click here for file

Additional file 2 **Clustal W Alignment of SSU RNA sequences from different *Plasmoidum spp.*** Hyphens indicate gaps. Colour scale from minimal consensus (dark blue) to exact consensus (red). Click here for file
