# Supplementary material for: Plasmodium vivax malaria in Mali: a study from three different regions
Source: Malar J. 2012 Dec 5;11:405. doi: 10.1186/1475-2875-11-405 (PMC3547733; doi:10.1186/1475-2875-11-405)
Supplement: Additional file 1 — Vivax malaria in Mali. Additional illustrative images of Giemsa-stained smear P. vivax positive samples from different regions. [file 1475-2875-11-405-S1.pdf]

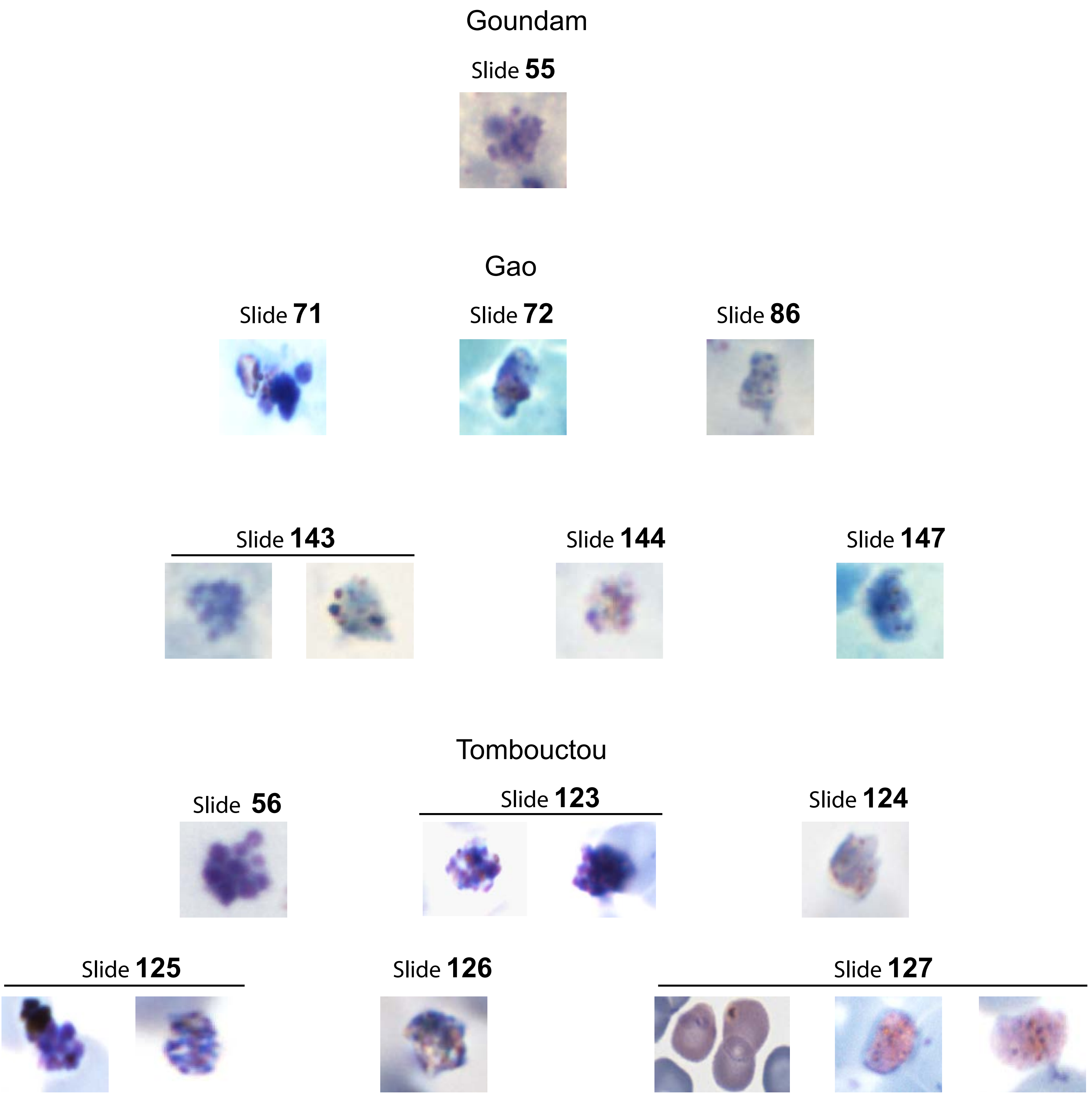
Goundam
Slide 55
Gao
Slide 71
Slide 72
Slide 86
Slide 143
Slide 144
Slide 147
Tombouctou
Slide 56
Slide 123
Slide 124
Slide 125
Slide 126
Slide 127
