# Supplementary material for: Plasmodium vivax malaria in Mali: a study from three different regions
Source: Malar J. 2012 Dec 5;11:405. doi: 10.1186/1475-2875-11-405 (PMC3547733; doi:10.1186/1475-2875-11-405)
Supplement: Additional file 2 — Clustal W Alignment of SSU RNA sequences from different Plasmoidum spp. Hyphens indicate gaps. Colour scale from minimal consensus (dark blue) to exact consensus (red). [file 1475-2875-11-405-S2.pdf]

```
Sequence Name         < Pos = 378
- +
Consensus             ------------GATTACTTCCAAGCCG----AAGCAAAGAAAGTCCTTAAATA--TGATGCAGT---AA-GAAAT-A-------TAAGGCTAAGATAT-GTACATTAAGTTGAAATGATG-TG--TATCAACTA--TGTGGATTAAGCTAGAAGCGAATCACTAGT
31 Sequences           380       390       400       410       420       430       440       450       460       470       480       490       500       510       520       530       540
Pknowlesi.seq         ACCACATCTAAGGAAGGCAGCAGGCGCG----TAAATTACCCAA-TTCTAAAGAAGAGAGGTAGTGACAA-GAAATAACA-AT-ACAAGGCCAA-TC-T-GGCTTTGTAATTGGAATGATGGGAATTTAAAACCTTCCCAAAATT-CAATTGGAGGGCAAGTCTGGT
Pmalariae.seq         ACCACATCTAAGGAAGGCAGCAGGCGCG----TAAATTACCCAA-TTCTAAAGAAGAGAGGTAGTGACAA-GAAATAACA-AT-GCAAGGCCAAATTTT-GGTTTTGCAATTGGAATGATGGGAATTTAAAACCTTCCCAGAAGG-CAATTGGAGGGCAAGTCTGGT
Povale.seq            ACCACATCTAAGGAAGGCAGCAGGCGCG----TAAATTACCCAA-TTCTAAAGAAGAGAGGTAGTGACAA-GAAATAACA-AT-ACAAGGCCATTTCAT-GGTTTTGTAATTGGAATGATGGGAATTTAAAACCTTCCCAAAATT-CAATTGGAGGGCAAGTCTGGT
Pyoelii.seq           ACCACATCTAAGGAAGGCAGCAGGCGCG----TAAATTACCCAAATTCTAAATAAGAGAGGTAGTGACAA-GAAATAACA-AT-ATAAGGCCAAATT-T-TGGTTTATAATTGGAATGATGGGAATTTAAAACCTTCCCAAAAAT-CAATTGGAGGGCAACTCTGGT
Slide 52_clone1.seq   ------------GATTACTTCCAAGCCG----AAGCAAAGAAAGTCCTTAAAAT--TGTTGCAGTTA-AAACG------------GGAGTTTAAGGCAA-CAACAGGGGCTTTAAAAT----TG--TATCAGTTA--TGTGGATTAAGCTAGAAGCGAATCACTAGT
Slide 53_clone1.seq   ------------GATTACTTCCAAGCCG----AAGCAAAGAAAGTCCTTACTTC--CAAGCCGAAGAAAGCAAAG------TCCTTACTTCCAAGCCGAAGCAAAGAAAGTCCTTACTTC--CAA-GCCGAAGCA---ATACCTCCAGCTGTTTGACAATCACTAGT
Slide 53_clone2.seq   ------------GATTACTTCCAAGCCG----AAGCAAAGAAAGTCCTTAAAAT--TGTTG-------------------------GAGTTTAAGGCAA-CAACAGGAGCGTTAAAAT----TG--TATCAGTTA--TGTGGATTAAGCTAGAAGCGAATCACTAGC
Slide 55_clone1.seq   ------------GATTACTTCCAAGCCG----AAGCAAAGAAAGTCCTTAGAAC--TTAGATA---------------------C-----CCAGGTAA-TTTGATCGGCTTGGAGG---------TATCAGTTA--TGTGGATTAAGCTAGAAGCGAATCACTAGT
Slide 56_clone1.seq   ------------GATTACTTCCAAGCCG----AAGCAAAGAAAGTCCTTA------------------AATCG-----------------GTGACGTAG-GCACAGTCGTTTTACAACGTCGTG--TATCAGTTA--TGTGGATTAAGCTAGAAGCGAATCACTAGT
Slide 61_clone1.seq   --------------------------CG----CTTCTAGCTTAA--TCCACATAACTGATACTTCGTATC-GACTTTGTGCGC-ATTTTGCTATTATGT-GTTCTTTTAATTAAAATGATTCTTTTTAAGGACTTTCTTTGCTTC-GGCTTGGAAGT
Slide 61_clone2.seq   ------------GATTACTTCCAAGCCG----AAGCAAAGAAAGTCCTTAAATCGGTGACGTA---------------------------TCAGCTAT-GTGGATTAAGCTAGAAG------G--TATCAGTTA--TGTGGATTAAGCTAGAAGCGAATCACTAGT
Slide 64_clone1.seq   --------------------------CG----CTTCTAGCTTAA--TCCACATAACTGATACCCT------GTTGTTGC-------CTTAAACTCCT-T-GCGTTTTAA-CTGCAACAATTTT----AAGGACTTTCTTTGCTTC-GGCTTGGAAGT
Slide 64_clone2.seq   ----------------------GATTCG----CTTCTAGCTTAA--TCCACATAACTGATAC---------GACGTTG----------TAAAACGAC-T-GTGCCT--A-CGTCACCGATTT-----AAGGACTTTCTTTGCTTC-GGCTTGGAAGTAATCACTAGT
Slide 64_clone3.seq   ------------GATTACTTCCAAGCCG----AAGCAAAGAAAGTCCTTAAAAT--TGTTGCAGTTA-AAACG-------------GAGTTTAAGGCAA-CAACAGGAGGTTTAAAAT----TG--TATCAGTTA--TGTGGATTAAGCTAGAAGCGAATCACTAGT
Slide 70_clone1.seq   ------------GATTACTTCCAAGCCG----AAGCAAAGAAAGTCCTTA------------------AATCG-----------------GTGACGTAG-GCACAGTCGTTTAA---------G--TATCAGTTA--TGTGGATTAAGCTAGAAGCGAATCACTAGT
Slide 70_clone2.seq   ------------GATTACTTCCAAGCCG----AAGCAAAGAAAGTCCTTAAAAC--TGTTGCAGTTA-AAACGTA---------AGGAGTTTAAGGCAA-CTACAGGAGCTTTAAAAT----TG--TATCAGTTA--TGTGGATTAAGCTAGAAGCGAATCACTAGT
Slide 71_clone1.seq   ---------------------CGATTCG----CTTCTAGCTTAA--TCCACATAACTGATACC------------TTA-----------GATACCCT-G-GTAATTTGATCGGCTTGGAAGTT----AAGGACTTTCTTTGCTTC-GGCTTGGAAGTAATCACTAGT
Slide 71_clone2.seq   ------------GATTACTTCCAAGCCG----AAGCAAAGAAAGTCCTTAAATCGGTGACGTAGGCACAGTCGTTTTACACGTCGTGCGTATCAGTTAT-GTGGATTAAGCTAGAAGCG----G--TATCAGTTA--TGTGGATTAAGCTAGAAGCGAATCACTAGT
Slide 71_clone3.seq   ------------GATTACTTCCAAGCCG----AAGCAAAGAAAGTCCTTAGAATA-TCAGGCAG-----------------------TATCAGTTAT-GTGGATTAAGCTAGAAGCG----G--TATCAGTTA--TGTGGATTAAGCTAGAAGCGAATCACTAGT
Slide 72_clone1.seq   ------------GATTACTTCCAAGCCG----AAGCAAAGAAAGTCCTTAGAAC--TTAGATA---------------------C-----CCAGGTAA-TTTGATCGGCTTGGAGG---------TATCAGTTA--TGTGGATTAAGCTAGAAGCGAATCACTAGT
Slide 72_clone2.seq   ----------------------GATTCG----CTTCTAGCTTAA--TCCACATAACTGATACCAC------GACGTTG----------TAAAACGAC-T-GTGCCT--A-CGTCACCGATTT-----AAGGACTTTCTTTGCTTC-GGCTTGGAAGTAATCACTAGT
Slide 83_clone1.seq   ----------------------GATTCG----CTTCTAGCTTAA--TCCACATAACTGATACTTCGTATC-GACTTTGTGCGC-ATTTTGCTATTATGT-GTTCTTTTAATTAAAATGATTCTTTTTAAGGACTTTCTTTGCTTC-GGCTTGGAAGTAATCACTAGT
Slide 86_clone1.seq   ------------GATTACTTCCAAGCCG----AAGCAAAGAAAGTCCTTAGTCC--T--TA-------AATCG-----------------GTGACGTAG-GCACAGTCATTTTACAACGTCGTGG-TATCAGTTA--TGTGGATTAAGCTAGAAGCGAATCACTAGT
SlideBrazil.seq       ----------------ACTTCCAAGCCG----AAGCAAAGAAAGTCCTTAAAAA---GAATCATTTTAATTAAAAGAACACATAATAG---CAAAATGC-GCACA--AAGTCGATACGAAGTATC-AGTTA------TGTGGATTAAGCTAGAAGCG
18S PVX_079693.seq    TTTCAAAGAATCGATATTTTAAGCAACG----CTTCTAGCTTAA--TCCACATAACTGATACTTCGTATC-GACTTTGTGCGC-ATTTTGCTATTATGT-GTTCTTTTAATTAAAATGATTCTTTTTAAGGACTTTCTTTGCTTC-GGCTTGGAAGT
18S PVX_088859.seq    ------AGTAACAAAATCTTCCATATGGTT--AAGGTAAAGAAGATTTTAAAAA---GAAACACTTTAATTAAAAAAACGCTTGATAGG--TAGGATGC-GCACAG-AAGCCGAAACCGTGATTG-GCTCAACCCCTTGTGGATCTAGCTAACGGCG--TTATT
18S PVX_096002.seq    ------AGTAACAAGGACTTCCATGCCG----AAGCAAAGAAAGTCCTTAAAAA---GAATCATTTTAATTAAAAGAACACATAATAG---CAAAATGC-GCACA--AAGTCGATACGAAGTATC-AGTTA------TGTGGATTAAGCTAGAAGCG--TTGCTT
18S PVX_097020.seq    ------AGTAACAAGGACTTCCAAGCCG----AAGCAAAGAAAGTCCTTAAAAA---GAATCATTTTAATTAAAAGAACACATAATAG---CAAAATGC-GCACA--AAGTCGATACGAAGTATC-AGTTA------TGTGGATTAAGCTAGAAGCG--TTGCTT
Pberghei.seq          GATATTTTTCAATGATACAACAAATATAGGAAATGTGTGTACAAGCTTCACATA-CAAATATAT----A---AGACAAGCCATTTTGCATTTAATATTTTATTTAAAAATTTATCTTTTTTTTAGACAAAAAAAA---ATAAATAAACATAAGAAAAATATATTAAC
Pcynomolgi.seq        ACCACATCTAAGGAAGGCAGCAGGCGCG----TAAATTACCCAA-TTCTAAAGAAGAGAGGTAGTGACAA-GAAATAACA-AT-ACAAGGCCAA-TC-T-GGCTTTGTAATTGGAATGATGGGAATTTAAAACCTTCCCAAAACT-CAATTGGAGGGCAAGTCTGGT
Pfalciparum.seq       ACCACATCTAAGGAAGGCAGCAGGCGCG----TAAATTACCCAA-TTCTAAAGAAGAGAGGTAGTGACAA-GAAATAACA-AT-GCAAGGCCAATTTTT-GGTTTTGTAATTGGAATGGTGGGAATTTAAAACCTTCCCAGAGTAACAATTGGAGGGCAAGTCTGGT
```
